# Editorial: Psychedelic sociality: Pharmacological and extrapharmacological perspectives

**DOI:** 10.3389/fphar.2022.979764

**Published:** 2022-07-22

**Authors:** Leor Roseman, Katrin H. Preller, Evgenia Fotiou, Michael J. Winkelman

**Affiliations:** ^1^ Centre for Psychedelic Research, Faculty of Medicine, Imperial College London, London, United Kingdom; ^2^ Pharmaco-Neuroimaging and Cognitive-Emotional Processing, Department of Psychiatry, Psychotherapy and Psychosomatics, Psychiatric Hospital, University of Zurich, Zürich, Switzerland; ^3^ Independent Researcher, Cleveland, OH, United States; ^4^ Retired, School of Human Evolution and Social Change, Arizona State University, Tempe, AZ, United States

**Keywords:** biopsychosocial, set and setting, culture, 5-HT2A receptor, social pharmacology, psilocybin, LSD, interdisciplinary research

The heralded psychedelic renaissance is currently at a new level where psychedelics are being accepted by the scientific community and the public. Medicalization and the ongoing introduction of market forces are imposing a trend in which psychedelic treatments are reduced to focus into strictly pharmacological and psychological effects on the self, rather than interactions with broader social context. Such narrowing of how psychedelic treatments are being conceived, used, and researched is a source of concern for those who understand that psychedelics’ therapeutic effects as also derived from socially and culturally meaningful elements. Alienation–the sense of isolation from others–and the mental health problems associated with it are on the rise. Consequently, there is not only a need for new therapies but also for a renewed social adhesion and a commitment to a more just and equal society. Psychedelics have a long history of bringing people together, facilitating intense shared experiences, and revitalizing cultures. This social dimension of use of psychedelics—*psychedelic sociality*—should be considered in the current mainstreaming, as therein lies their potential to support change in individual therapy and beyond.

This multidisciplinary Research Topic of *psychedelic sociality* invited scholars to discuss these Research Topic through empirical research, reviews, perspectives, and theoretical papers. Overall, 21 papers were accepted to this Research Topic, covering different sections of Frontiers (Neuropharmacology, Psychopharmacology, Ethnopharmacology, Personality and Social Psychology, and Consciousness Research). We are especially proud of the broad scope of this Research Topic and the diversity of disciplines represented in it. We believe that a beneficial mainstreaming of psychedelics requires going beyond the boundaries of disciplinary orientation. Interdisciplinary integration is necessary for the paradigmatic shift in mental health that many are yearning for: a shift from a narrow biomedical model to an expanded biopsychosocial model that emphasizes the interplay between biological, psychological, sociopolitical and environmental factors in mental health. The centrality of the experience and set and setting in psychedelic research^1^ is an invitation to transcend some of the boundaries between the natural sciences and the humanities (see Langlitz et al.). The biopsychosocial model is especially relevant to psychedelic research because to answer questions regarding how psychedelics work, we must incorporate different levels of inquiry–from receptors to persons to culture.

Psychedelic pharmacological interventions are influenced by contextual factors such as social relations, language, books, theories, symbols, music, and beliefs. Notably, this is a reciprocal relationship, and the psychedelic effects can, in turn, change context, e.g., enhance openness, facilitate the creation of new music, modify social relationships, change personalities, and revitalize cultures. Psychedelic communities, Western and indigenous, each have their own musical and artistic expression, suggesting that the communal use of psychedelics intensifies cultural modes of communication. The context-sensitive role of 5-HT2A receptors in sociality is also noticeable in that they can be anxiogenic when trust is compromised but rewarding within secure contexts, and hence enhancing sociality by encouraging healthy contexts.

These facts amplify the relevancy of the human sciences for psychedelic research, and vice versa, as what makes humans so unusual are two interrelated aspects: our capacity for culture (especially cumulative technological and social knowledge); and our sociality, an unprecedented and unparalleled ability to live in large groups of unrelated people. The human capacity for sociality involved a Darwinian coevolution of culture and genes (Richerson and Boyd, 2008); our biology evolved to create our capacity for culture and culture shaped our biological evolution. As psychedelics are notoriously context-dependent, one can speculate that their effects on the 5-HT2A receptor function could have played a crucial role in the selection for this unique human capacity. The activation of the 5-HT2A receptor intensifies the influence of social relations, stories, beliefs, and symbols by incorporating them with our sensory experience, making them more tangible, hence enhancing their meaning and increasing our capacity for sociality. This suggestion is also supported by a number of human fMRI studies from various labs, showing increased functional connectivity between sensory regions (especially visual) and higher level regions (Roseman et al., 2014; Carhart-Harris et al., 2016; Tagliazucchi et al., 2016; Müller et al., 2018; Preller et al., 2018; Mason et al., 2020; Preller et al., 2020).

In this Research Topic, Arce and Winkelman theorize that psychedelics enhanced human evolution in the cultural niche as hominins became dependent on shared cultural models for basic survival. The sociality-amplifying effects of psilocybin and its ability to enhance openness and increase novel representations and flexibility in cognitive processes could have created considerable fitness differences among ancestral hominin populations. Individual and eventually population differences in abilities to respond to these effects would have provided genetic variation upon which selection pressures could act in favour of those with the capacities for sociality and cognition stimulated by psilocybin. Their paper provides a summary of the role of 5-HT2A activation and reviews clinical research showing how psychedelics promote sociality through managing psychological stress, improving interpersonal relations, facilitating of collective relations, and enhancing group decision making.

The influence set and setting have on psychedelic experiences provides some of the most convincing arguments for *psychedelic sociality*. The recent book *American Trip* by Ido Hartogsohn (2020), reviewed here by Lansky, presents an historical analysis that clearly shows how different set and setting factors embodied in research orientations led to different psychedelic experiences and different understandings of psychedelics’ action. Some papers in this Research Topic are also examining how social and cultural context affect the psychedelic experience. Pontual et al. and Pontual et al. presents results from a large online cross-cultural study used in development of the Setting Questionnaire for the Ayahuasca Experience (SQAE), and showed its relation to the quality of the psychedelic experience. The SQAE has six dimensions (Leadership, Decoration, Infrastructure, Comfort, Instruction, and Social). High ratings on the SQAE were associated with low ratings of challenging experiences and high ratings of mystical experiences in three different ayahuasca practices (Santo Daime, UDV, and neoshamanic).

A similar observation is presented here by Hartogsohn, who researched the migration of Santo Daime ceremonies to online zooms during the COVID-19 pandemic. While online ceremonies created new possible global connections, the lack of immediate and embodied social context was also associated with reduced intensity of the experience. Murphy et al. present similar results in a seminal controlled clinical trial comparing psilocybin-assisted therapy for depression versus a control group with escitalopram. They found pre-session therapeutic alliance and rapport predicted the intensity of the acute psychedelic experience (emotional breakthrough and the mystical experience); these in turn were associated with improvements in depressive symptom severity 6 weeks after the experience. The above studies show that social bonding and trust are vital components for effective and safe psychedelic therapy.

It is important to recognize that it is not only the immediate interpersonal context that interacts with psychedelics, but also the cultural context. In this Research Topic, de Mori, using ethnographic data, shows that the attribution of efficacy in diverse psychedelic practices differs based on cultural priors, ideologies or biases. Along similar lines, Dupuis expands on his previous ethnographic work to show that psychedelic-induced belief transmission is a process that oscillates between adherence and doubt. This oscillation strongly mobilizes the reflexivity and agentivity of the recipient and can paradoxically enhance belief transmission. Furthermore, as the phenomenological content of the experience itself is influenced by cultural priors, it can strengthen the newly acquired belief through “experiential verification.” When stories become visions, they also become more convincing.

Some central qualities of psychedelic experiences exhibit sociality. In some contexts, psychedelics acutely increase relational feelings of connectedness with nature, other humans, or with spirituality (Forstmann and Sagioglou, 2017; Watts et al., 2017; Yaden et al., 2019; Kettner et al., 2021). Psychedelics can produce an animistic mindset, where the natural world is humanized, personalized and socialized with human traits (sentience, relationality, intentionality, cooperation, intelligence) (Winkelman, 2013). In this Research Topic, Michael et al. presents a microphenomenological analysis of DMT experiences. Most experiential reports described an encounter with “sentient beings” that were experienced as “other.” The encounters were rich in different features, such as the entities’ appearances, demeanour, roles, function, and included communication and interaction. The complex interactive social imagery likely reflects psychedelic action on the 5-HT2A receptors; psychedelic entity experiences also reflect activation of innate cognitive modules directly related to unique human capacities for sociality (Winkelman, 2018).

Diverse findings indicate that psychedelics promote prosocial effects. In this Research Topic, Holze et al. show in a placebo-controlled trial that LSD acutely increases empathy and plasma oxytocin levels. Weiss et al. online prospective study found psychedelics increased social connectedness and agreeableness. Luoma and Lear review evidence that MDMA-assisted therapy can successfully treat Social Anxiety Disorder, reminding us of the empathogenic effects of the serotonergic system in general. Evens et al. found in an online retrospective study that during the first wave of COVID-19 (April to August 2020), the quality of the psychedelic experience became more prosocial. Their finding reflects some of the zeitgeist of the first wave, when liminality and global communitas were experienced, in which people found a momentary sense of solidarity and liberation from day-to-day social hierarchies and tensions. Another study in this Research Topic suggests that regular psychedelic use is associated with better coping with COVID-19 social confinements (Révész et al.). However, causality cannot be inferred from this study as other factors were different for psychedelic users, such as more substantial social support during the pandemic. Newson et al. study here with a preregistered online survey on people who attend raves and “free-parties” found psychedelics and dancing lead to personal transformation and that the acute experience of awe mediated this transformation. In turn, both awe and personal transformation increase bonding to fellow ravers and prosocial behaviour (donation to a rave charity). Similarly, Murphy et al. psilocybin clinical trial mentioned above found not only that the therapeutic alliance facilitates stronger emotional breakthroughs, but also that emotional breakthroughs enhance the therapeutic alliance in return. Shared intense emotional experiences can promote social bonding, whether one is a patient or a raver.

Markopoulos et al. presents a compelling summary of 20 clinical and experimental trials since 2008 that show psychedelics’ prosocial and empathogenic effects. Drawing from these findings and LSD trials from the 60s’ that showed that psychedelic-assisted therapy helped children with Autism Spectrum Disorder (ASD), they argue for the potential use of psychedelics in the treatment of ASD. Yet, they note, that some children had adverse reactions which led to increased anxiety and aggression, and even to self-harm. Importantly, in these trials “greater improvements were observed when the therapist was more actively involved with the children; when they were given the possibility to experience meaningful interpersonal psychotherapeutic interactions; and when the settings were free of artificial or experimental restrictions.” Once again, we see that the reaction to the drug is modulated by contextual factors. Consequently, the prosocial effects cannot be attributed to the drug alone but to drug and context interactions.

This point is illustrated in several papers in this Research Topic. Pace and Devenot critique universal claims that psychedelics–in their pharmacological essence—necessarily improve society, increase environmental concern and promote liberal politics. They present examples of right-wing psychedelia in which conservative ideologies assimilate psychedelic experiences of interconnection but amplify authoritarian worldviews. They suggest that psychedelics are “politically pluripotent” in that the changes they induce are based on the political setting. Langlitz et al. also raise caution against universal claims about psychedelics’ moral psychopharmacology, which requires an interdisciplinary inquiry to prevent naïve interpretations of results: “While members of the American counterculture used LSD, mescaline, and psilocybin as psychopharmacological tools to liberate individuals from the ills of their society, Huichol youth ingested peyote buttons to become full members of their own society, and Native American Church worshippers consumed peyote to foster indigenous resistance to North American colonialism. In the 1960s, psychedelics were taken to experience a mystical union that users claimed fostered a sense of universal love, but anthropologists have also described Amazonian societies that used them in rituals to prepare for violent intergroup conflict.”

Roseman and Karkabi—in a sequel paper to Roseman et al. (2021)—made similar critical observations regarding politically pluripotent dynamics while studying ayahuasca groups in which Israeli and Palestinians drink together in rituals organized by Israelis. While the collective experience of “oneness” and interconnectedness can create strong social bonds between Israelis and Palestinians, it does not necessarily produce more equality and justice. The “irony in harmony” is that too much focus on harmony can bypass political tensions, and so stabilize the hegemonic structures in which Israelis subjugate Palestinians. When harmony is the main goal of the status quo, any mention of injustice can be silenced to prevent disharmony. Prosocial effects and increased group cohesion are not inherently just, and can sometimes be in service of hierarchies, unequal social structures, and violence. Yet, the authors also identify unique revelatory events which momentarily rupture the harmony of the ritual by revealing agonizing visions of Palestinian trauma which are not aligned with the hegemonic Israeli narrative. Such events can ignite a liberative procedure in which the subjects attempt to change the social structure. The authors argue that such revelatory events can ignite resistance towards the status-quo and can activate the subject with revolutionary hope, acting towards a more egalitarian structure.

Recognition of cultural and social influences on psychedelics’ mechanisms of action reveals a complexity of mechanisms that raise caution with oversimplified and hyped claims about psychedelics. Studying psychedelics only through the dominant paradigms in psychiatry and psychology ignores many of their functions. While integrating psychedelics into such paradigms is necessary for their mainstream acceptance, this comes with a risk that psychedelics will be assimilated into these paradigms instead of changing them, and consequently restricting psychedelic practices and the science of psychedelics. The simplified mainstream narratives ignore broader knowledge and perspectives about psychedelic use and mechanism of action. The seemingly controversial argument that psychedelics’ therapeutic efficacy is dependent on suggestibility, social context, and cultural priors challenges traditional psychopharmacological views. But for anyone who seeks universal scientific truths about psychedelics, these might be their principal mechanism of action, a non-specific amplification of context, which is strongly related to sociality, especially interpersonal relations and cultural setting.

Novel theoretical orientations that incorporate biopsychosocial notions are therefore required. Lepow et al. presents these here in a testable hypothesis in which psychedelics induce a critical period plasticity that “remove the brakes on adult neuroplasticity, inducing a state similar to that of neurodevelopment.” It is suggested that this enhanced sensitivity to environmental input and increased relearning capacity are then utilized in the therapeutic procedure to create enduring effects.

Future studies need to expand beyond conventional clinical research into process-oriented research, such as examining how mediating variables produce different outcomes, and reporting clinical case studies. The extrapharmacological complexity of psychedelic effects means that evidence-based medicine has its (double-)blind spots, as it provides us with controls to eliminate the very conditions that psychedelics exploit—the interpersonal dynamics and placebo and expectancy effects.

This can miss the underlying mechanism of psychosociotheraputic processes engaged or released by psychedeics. Therefore, another potential research avenue into *psychedelic sociality* is cultural-controlled trials (Wallace, 1959) which experimentally examine cultural differences in trial outcomes. Such trials can even incorporate biological measures such as oxytocin levels to assess how context influences the most basic sociopharmacological dynamics. Other studies can investigate interpersonal dynamics as introduced in this Research Topic in Wagner on evaluating MDMA couples therapy.

To conduct biopsychosocial psychedelic research, interdisciplinary collaborations are encouraged. Such collaborations are healthy for psychedelic research, but more crucially, they might impact science at large. In the current state of academic affairs, biologists, psychologists and social scientists are studying the same human without clearly communicating their findings to each other. Psychedelic research can serve as a glue that integrates interdisciplinary knowledge on mind, body and culture, and so reveal what is unique about humans.

## Footnotes

1 All pharmacological interventions are dependent on extrapharmacological factors to some extent, exemplified in the placebo response, yet this interaction seems much more pronounced with serotonergic psychedelics.
